# Sparse regression, classification, and microbial network estimation in QIIME 2 with q2-classo and q2-gglasso

**Published:** 2026-04-16

**Authors:** Oleg Vlasovets, Fabian Schaipp, Léo Simpson, Evan Bolyen, J. Gregory Caporaso, Christian L. Müller

**Affiliations:** 1 Computational Health Center, Helmholtz Munich, Neuherberg, Germany; 2 Ludwig-Maximilians-Universität München, Department/Faculty, München, Germany; 3 Technical University of Munich, Department/School, München, Germany; 4 Albert-Ludwigs-Universität Freiburg, Department/Faculty, Freiburg, Germany; 5 Pathogen and Microbiome Institute, Northern Arizona University, Flagstaff, AZ, USA; 6 Center for Computational Mathematics, Flatiron Institute, New York, NY, USA

**Keywords:** QIIME 2, high-dimensional statistics, compositional data analysis, microbial networks, sparse regression, graphical lasso

## Abstract

**Motivation:**

Statistical analysis of microbial count data derived from 16S rRNA or metagenomics sequencing poses unique challenges due to the sparse, compositional, and high-dimensional nature of the data. While QIIME 2 already provides many tools for data pre-processing and analysis, plugins for statistical regression, classification, and microbial network estimation tailored to compositional count data are relatively scarce.

**Results:**

We present q2-classo and q2-gglasso, two novel QIIME 2 plugins that implement penalized regression, classification, and graphical modeling approaches for microbial compositional data. q2-classo enables the prediction of a continuous or binary outcome of interest using compositional microbiome data as predictors. Both sparse log-contrast regression and classification, as well as tree-aggregated log-contrast models are available. q2-gglasso enables the estimation of taxon-taxon association networks through sparse graphical model estimation, such as, e.g., the SPIEC-EASI framework, as well as adaptive and latent graphical models. The latent model can decompose taxon-taxon associations into a sparse direct interaction matrix and a latent (low-rank) matrix which enables robust principal component embedding of a data set. Within the QIIME 2 ecosystem we demonstrate their application on the Atacama soil microbiome dataset, illustrating robust model selection, classification, and microbial network estimation with covariates and latent factors.

**Availability:**

The software is freely available under the BSD-3-Clause License. Source code is available at https://github.com/bio-datascience/q2-gglasso and https://github.com/bio-datascience/q2-classo-latest, with installation through QIIME 2 and Docker.

**Contact:**

oleg.vlasovets@helmholtz-munich.de

## Introduction

Microbial abundance data, derived from high-throughput amplicon or metagenomics sequencing experiments and subsequent dedicated pre-processing [5], comprise microbial counts in form of amplicon sequence variants (ASVs), operational taxonomic units (OTUs), or metagenomics-derived OTUs (mOTUs). These counts are characterized by an excess number of zeros and carry only compositional (or relative abundance) information [15]. Moreover, in a typical microbiome dataset the number of microbial features is larger than the number of available samples (i.e., data are in the “high-dimensional” regime), making downstream statistical estimation tasks, such as differential abundance testing [26], regression [20], or covariance and network estimation [18], challenging.

**Fig. 1.**
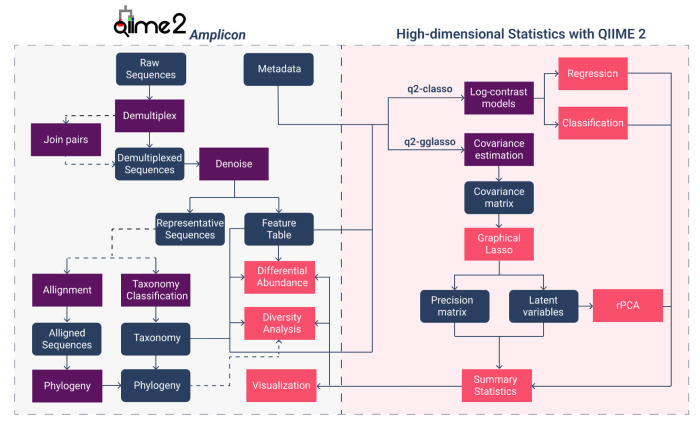
QIIME 2 workflow and its high-dimensional statistics extension. The left panel illustrates typical amplicon processing steps in QIIME 2. Feature Table, Taxonomy objects and available Metadata serve as input to the high-dimensional statistics plugins q2-classo and q2-gglasso (right).

In this contribution, we introduce q2-classo and q2-gglasso, two novel QIIME 2 plugins, that implement principled methods for (high-dimensional) statistical regression, classification, and network estimation for microbial compositional data. This enables microbiome researchers easy access to these tools within the QIIME 2 ecosystem, an open-source microbiome bioinformatics platform, recognized for its robust functionality, reproducibility through automatically tracked data provenance, extensibility through a module-based plugin architecture, and broad user and developer community [4]. While QIIME 2 already comprises a number of plugins for pre-processing raw sequences [5], diversity analysis [4, 29], community structure investigation [4, 21, 23], and differential abundance testing [12, 19], our plugins complement existing statistical approaches for regression, classification, and network estimation (see Fig. 1). For instance, the popular q2-sample-classifier [3] plugin offers standard machine learning methods such as Ridge and Lasso regression, but does not provide log-contrast regression models tailored to compositional data. q2-longitudinal [3] supports linear mixed-effects models and paired- sample comparisons for low-dimensional time-series. songbird [26] uses multinomial regression specifically for differential abundance testing. Qurro [11] enables the visualization of quantitative ranks and ratios, often in the form of log-contrasts or log-ratios, but cannot explicitly build combinations of log-ratios in a data-driven manner. In the context of microbial network estimation, the currently only available tool is q2-SCNIC [31]. SCNIC (Sparse Correlation Network Investigation for Compositional data) constructs standard pairwise correlation networks from feature tables using metrics such as SparCC [14], detects co-occurrence modules, and allows for visualization of correlated features and modules.

We next describe the key objectives of the two plugins, outline their mathematical underpinning, and their embedding within QIIME 2. We also illustrate a practical use case on the Atacama soil microbiome dataset.

## Methods and implementation

### Log-contrast regression and classification for microbial relative abundances with q2-classo

q2-classo enables the prediction of a continuous or binary outcome of interest from high-dimensional microbial abundance data. The underlying statistical model is the so-called log-contrast model, as introduced in [1] for regression, where an outcome of interest, e.g., an environmental covariate or the disease status of patient, is implicitly modeled as a linear combination of log-ratios of individual compositional parts (e.g., microbial relative abundances). This model has been extended to sparse (penalized) estimation in [20, 32] when more features (taxa) are available than samples. Since then, the model and its underlying statistical estimation has been further refined to allow for robust regression [7, 25], joint estimation of noise [7], taxonomic tree-aggregated regression (trac [2]), and classification [33]. q2-classo inherits these log-contrast models from the c-lasso package [33] and makes them available in QIIME 2.

Figure 1 shows a typical QIIME 2 amplicon sequencing data analysis pipeline and its connection to q2-classo. While a typical analysis comprises multiple functionalities, the QIIME 2 artifacts Feature Table[Frequency]  and FeatureData[Taxonomy] , as well as Metadata (containing outcomes of interests) serve as direct inputs to q2-classo. Prior to model fitting, q2-classo applies a centered log-ratio transform to the feature counts to account for the compositional nature of the data. Broadly, three model classes are available: (i) sparse log-contrast regression for continuous outcomes, (ii) sparse log-contrast classification for binary outcomes, and (iii) tree-aggregated log-contrast (trac) models which can incorporate taxonomic information. To guard against overfitting, q2-classo provides three model selection strategies: a theoretically-derived fixed penalty [32, 7], *k-*fold cross-validation, and stability selection [24, 7, 16]. After model estimation, q2-classo stores the model output, including selected taxa, their effect sizes, and model selection curves, in QIIME 2 .qza/.qzv files, which also enable visualizations for interactive inspection.

### Microbial network estimation with q2-gglasso

q2-gglasso enables the estimation of taxon-taxon association networks via the application of so-called graphical modeling techniques. The principle idea is to estimate a *sparse* inverse covariance (or *precision*) matrix from a given sample covariance matrix of the (microbial) features using a penalized likelihood [9], also referred to as graphical lasso [13]. The non-zero coefficients of the estimated inverse covariance matrix are related to partial correlations and can be interpreted as associations between the different features (taxa) and summarized as network. Important extensions of this model include the estimation of joint graphical models across multiple data sets [8] and latent graphical models [6]. The latter approach estimates both a sparse inverse covariance and a dense latent (low-rank) matrix that can adjust for hidden confounders and biases. The low-rank matrix, in turn, can then be used for robust principal component analysis (rPCA), similar in spirit to q2-deicode [22]. In the microbiome context, graphical modeling has been used in SPIEC-EASI (SParse InversE Covariance Estimation for Ecological Association Inference) [18, 17], gCODA [10], and others (see e.g., [28] for an overview). q2-gglasso inherits these network estimation schemes through the GGLasso package [30], an efficient Python implementation of a wide range of sparse graphical models, and makes them available in the QIIME 2 ecosystem.

As shown in Fig. 1, q2-gglasso takes as primary input Feature Table[Frequency]  and estimates taxon-taxon association networks via graphical modeling techniques. To address the compositional nature of the data, the centered log-ratio (clr) or a modified clr (mclr) transformation [34] is applied prior to covariance estimation. The following major graphical model classes are available: (i) standard graphical lasso [13], which recovers a sparse precision matrix representing conditional dependence structure among taxa — analogous to the SPIEC-EASI framework [18]; (ii) multimodal graphical lasso, which enables combined estimation of taxa–covariate associations (through adaptive penalization of the different modalities); and (iii) a sparse+low-rank model [6, 17] that decomposes the precision matrix into a sparse direct interaction component and a low-rank latent factor component, enabling latent variable correction and robust PCA of the microbiome data [17]. After model estimation, q2-gglasso stores the model output, including the estimated precision matrix, network summary statistics, low-rank components, and interactive network and heatmap visualizations, in QIIME 2 .qza/.qzv files for further visualizations and interactive inspection.

### Use case on Atacama soil dataset

We illustrate the functionality of both plugins by focusing on a subset 13 highly abundant taxa across 50 samples of the Atacama soil microbiome dataset [27]. To illustrate log-contrast regression with q2-classo, we predict average soil temperature as a continuous outcome. For classification, we considered vegetation type as a binary outcome. For both prediction tasks, stability selection consistently identified a small set of predictive taxa for the respective outcome.

To illustrate network estimation with q2-gglasso, we used the same subset of data to first estimate a sparse inverse covariance matrix to identify the partial correlation structure among microbial taxa. We showcase how to include the available covariate information and learn joint partial correlations over taxa *and* environmental covariates. Finally, we also learned a latent graphical model on the taxon data, yielding a sparse and a low-rank component. We finally show that the *inferred* latent components from the taxon data alone correlate well with the key environmental covariates. All estimated networks are visualized as interactive heatmaps within the QIIME 2 framework.

The detailed tutorial and reproducible pipeline examples are available in the online documentation at https://vlasovets.github.io/q2-hdstats-docs/intro.html.

### Availability and Implementation

Both plugins are Python implementations integrated into QIIME 2.

Software availability:

- q2-gglasso: library.qiime2.org/.../q2-gglasso
- q2-classo: library.qiime2.org/.../q2-classo-latest
- License: BSD-3-Clause

#### Data availability:

The Atacama soil microbiome dataset [27] is available from the European Nucleotide Archive (ERP019482). Processed count tables, covariates, and SILVA-based taxonomic annotations are also provided.

## Conclusion

q2-classo and q2-gglasso provide capabilities for compositionally-aware sparse regression, classification, and microbial network estimation directly within QIIME 2. Making these state-of-the-art high-dimensional statistics tools available in the QIIME 2 ecosystem allows, in turn, easy integration with existing analysis workflows, automated provenance tracking ensuring transparency and reproducibility, and accessibility to a broad user base.
